# Association between Dietary Niacin Intake and Nonalcoholic Fatty Liver Disease: NHANES 2003–2018

**DOI:** 10.3390/nu15194128

**Published:** 2023-09-25

**Authors:** Jie Pan, Yuhua Hu, Nengzhi Pang, Lili Yang

**Affiliations:** 1 Department of Nutrition, Guangdong Provincial Key Laboratory of Food, Nutrition and Health, School of Public Health, Sun Yat-sen University, Guangzhou 510080, China; panj39@mail2.sysu.edu.cn (J.P.);; 2 School of Public Health, Southeast University, Nanjing 210009, China

**Keywords:** dietary niacin, nonalcoholic fatty liver disease, NHANES

## Abstract

Evidence regarding the association between dietary niacin intake and nonalcoholic fatty liver disease (NAFLD) is limited. The objective of this study was to examine the association of dietary niacin intake with NAFLD. Subjects aged 20 years and older who participated in the National Health and Nutrition Examination Survey (NHANES) 2003–2018 were included in this study. Dietary niacin intake was assessed by two 24-h dietary recalls. NAFLD was defined using the United States fatty liver index (US-FLI). Weighted logistic regression models and restricted cubic splines were used to examine the association between dietary niacin and NAFLD. Of the 12,355 participants in this study, 4378 had NAFLD. There is no evident nonlinear relationship between dietary niacin intake and the presence of NAFLD in the restricted cubic spline regression (*p*_overall_ < 0.001; *p*_non-linearity_ = 0.068). The multivariable-adjusted odds ratios (ORs) and 95% confidence intervals (CIs) for NAFLD were 0.84 (0.68–1.03), 0.80 (0.65–0.97), and 0.69 (0.55–0.85), respectively, when comparing the second, third, and fourth quartiles of niacin intake levels to the lowest quartile (*p*_trend_ = 0.001). Stratified analysis revealed that the effect of niacin intake on NAFLD varied in the group with or without hypertension (*p*_interaction_ = 0.033). In conclusion, our results indicate that higher dietary niacin intake may be associated with a lower likelihood of NAFLD.

## 1. Introduction

Nonalcoholic fatty liver disease (NAFLD) is characterized by the presence of hepatic steatosis (≥5%) without significant alcohol consumption and other known causes of liver fat accumulation [1]. It has emerged as a public health challenge, with a prevalence of 38.0% during 2016–2019, and is poised to become the primary cause of chronic liver disease globally [2,3,4]. NAFLD is currently known as metabolic-dysfunction-associated fatty liver disease (MAFLD) under new nomenclature [5]. The increasing incidence of metabolic risk factors and the aging population is expected to more than double the burden of advanced NAFLD-related disease over the period 2016–2030 [3,6,7]. Thus, the prioritization of NAFLD prevention and treatment holds significant importance, encompassing both clinical and population-level considerations.

While pharmacological treatments for NAFLD are currently unavailable, lifestyle modifications are vital interventions. Given the influence of diet on NAFLD, targeted dietary strategies are essential. Niacin, also known as vitamin B_3_, serves as a nutritional precursor for both nicotinamide adenine dinucleotide (NAD) and nicotinamide adenine dinucleotide phosphate (NADP), which play crucial roles in cell metabolic reactions, energy metabolism, and redox reactions [8,9]. Animal studies have shown supplementation with NAD^+^ precursors could be a potential therapeutic strategy for the prevention and treatment of NAFLD [10,11]. Previous clinical research has indicated that niacin can effectively increase NAD^+^ levels in humans and improve fatty liver in individuals with mitochondrial myopathy [12]. Additionally, a lifestyle intervention study suggested that high dietary niacin intake may have a beneficial effect on reducing liver fat [13].

However, to date, the relationship between dietary niacin intake and NAFLD in the general population remains unclear. Therefore, our present study aimed to examine the association of dietary niacin intake with NAFLD using a nationally representative sample of United States (US) adults.

## 2. Materials and Methods

### 2.1. Study Design and Participants

The National Health and Nutrition Examination Survey (NHANES) is conducted to evaluate the health and nutritional status of the US population. Using a sophisticated multistage probability sampling design, the NHANES gathers information on the civilian population of the US through a two-year cycle [14]. The present cross-sectional study used NHANES data from 2003 to 2018, comprising US adults aged 20 years and above who took part in the survey. According to the presence or absence of NAFLD, they were divided into NAFLD group and non-NAFLD group.

### 2.2. Measurement of Dietary Niacin Intake

The dietary interview component, known as What We Eat in America (WWEIA), is carried out through collaboration between the US Department of Agriculture (USDA) and the US Department of Health and Human Services (DHHS). Two 24 h dietary recall interviews are administered to all eligible NHANES participants to report the types and quantities of foods they consumed in the 24 h before the interview (from midnight to midnight). These interviews aim to collect information regarding the quantities and varieties of foods individuals consumed in the 24 h preceding the interview, encompassing the time from midnight to midnight. A dietary recall was conducted face-to-face at the Mobile Examination Center (MEC), and the second recall took place through a telephone interview around 3 to 10 days subsequent to the initial recall. The USDA’s Food and Nutrient Database for Dietary Studies (FNDDS) was utilized to calculate the nutrients and food components in all food items [15]. The dataset on total nutrient intakes provided a summary record of the nutrient intake for each individual. In this study, the daily niacin intake of participants was determined by calculating the average of their two dietary recalls.

### 2.3. Definition of NAFLD

The definition of fatty liver was established based on the United States fatty liver index (US-FLI), which is computed in the following manner: US-FLI = e^y^/(1+e^y^) × 100, where y = −0.8073 × non-Hispanic black + 0.3458 × Mexican American + 0.0093 × age + 0.6151 × log_e_ (gamma glutamyltransferase) + 0.0249 × waist circumference + 1.1792 × log_e_ (insulin) + 0.8242 × log_e_ (glucose) − 14.7812. The values for ‘non-Hispanic black’ and ‘Mexican American’ are assigned as 1 if the participant belongs to that ethnicity and 0 if they do not belong to that ethnicity [16]. The definition of fatty liver was determined by selecting a score of US-FLI ≥ 30, according to the recommended value [17,18,19,20]. NAFLD is characterized by having a score of ≥30 on the US-FLI assessment, while excluding any other established factors of chronic liver disease. These factors encompass viral hepatitis (recognized by the presence of positive markers such as hepatitis B surface antigen, hepatitis C antibody, or hepatitis C RNA) as well as substantial alcohol intake (≥2 drinks per day for men or ≥1 drink per day for women).

### 2.4. Assessment of Covariates

In this study, age was classified into three groups: ≤39, 40–59, and ≥60 years. The race/ethnicity of the participants was categorized as Mexican American, non-Hispanic white, non-Hispanic black, and other. Education levels were categorized as less than high school, high school, and some college or above. Family income–poverty ratio was grouped into three categories: less than 1.0, between 1.0 and 3.0, and greater than 3.0. Smoking status was divided into never smokers (defined as having smoked less than 100 cigarettes in their lifetime), current smokers (defined as having smoked more than 100 cigarettes in their lifetime), and former smokers (defined as having smoked more than 100 cigarettes in their lifetime and having quit smoking). Participants were classified as physically active if they engaged in at least 150 min of moderate to vigorous physical activity per week according to the Centers for Disease Control and Prevention Physical Activity Guidelines for Americans, otherwise they were considered physically inactive. Body mass index (BMI) was categorized as <30.0 and ≥30.0. Information on a physician-diagnosed history of hypertension, high cholesterol, and diabetes was obtained through self-reporting.

### 2.5. Statistical Analysis

In this study, all analyses take into account the complex sampling design of NHANES. This means that sample weights, strata, and primary sampling units are incorporated to ensure accurate representation of the population. Data are presented as unweighted frequencies (weighted percentages) for categorical variables and as medians (interquartile range) for continuous variables. Differences between two groups were compared using the Wilcoxon rank-sum test for continuous variables that followed a non-normal distribution. On the other hand, the chi-squared test with the Rao–Scott second-order correction was employed to compare differences between two groups for categorical variables. We used weighted logistic regression models to determine odds ratio (OR) and 95% confidence interval (CI) in order to examine the association between dietary niacin intake and NAFLD. We constructed two models for analysis. Model 1 adjusted for age and gender. In Model 2, we further adjusted for race/ethnicity, education levels, family income–poverty ratio, smoking status, physical activity, body mass index, total energy intake, hypertension, high cholesterol, and diabetes. To examine the linear trend, we generated a continuous variable by assigning the median value to each category.

Restricted cubic spline analysis was used to examine the nonlinear associations between dietary niacin intake and NAFLD, introducing four knots positioned at the 5th, 35th, 65th, and 95th percentiles. The likelihood ratio test was conducted to evaluate nonlinearity. We further stratified the analyses by age, gender, race/ethnicity, education levels, family income–poverty ratio, smoking status, physical activity, BMI, hypertension, high cholesterol, and diabetes. Age was categorized as <60 years or ≥60 years, gender as male or female, race/ethnicity as non-Hispanic white or other, education levels as ≤high school or some college or above, family income–poverty ratio as ≤3 or >3, smoking status as never, former, or current, physical activity as active or inactive, BMI as <30.0 or ≥30.0, hypertension as yes or no, high cholesterol as yes or no, and diabetes as yes or no. The significance of the interactions was estimated using the *p* values for the production terms between dietary niacin intake and the stratified factors.

During the NHANES data collection period, there were changes in the laboratory techniques used to quantify glucose, insulin, alanine aminotransferase (ALT), asparate aminotransferase (AST), and gamma glutamyltransferase (GGT) concentrations, and we applied regression equations suggested by the NCHS to align those values over time. Covariates with missing values were imputed using the multiple imputation by chained equations method. All statistical analyses were performed using R software (version 4.2.3), with a two-sided *p* < 0.05 as the threshold for statistical significance.

## 3. Results

### 3.1. Selection of Study Population

**Figure 1:**
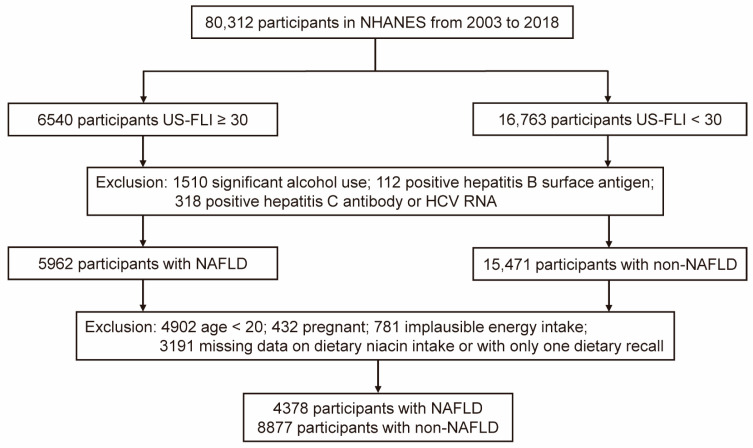
Flow chart illustrating selection of the study population in NHANES from 2003 to 2018.

Among the 80,312 participants in NHANES from 2003 to 2018, 6540 had a US-FLI greater than 30 and 16,763 had a US-FLI less than 30. After excluding participants with significant alcohol use, positive hepatitis B surface antigens, positive hepatitis C antibodies, or hepatitis RNA, 5962 participants had NAFLD and 15,471 participants with non-NAFLD. We further excluded participants who were younger than 20 years, pregnant, had missing data on dietary niacin intake or with only one dietary recall, or had implausible energy intake (<600 or >3500 kcal/day for women and <800 or >4200 kcal/day for men) [21]. In the end, 4378 participants with NAFLD and 8877 non-NAFLD participants were included in the analysis. A detailed description of the selection process for the study population can be found in Figure 1.

Abbreviations: NHANES, National Health and Nutrition Examination Survey; US-FLI, United States—fatty liver index; HCV, hepatitis C virus; and NAFLD, nonalcoholic fatty liver disease.

### 3.2. Baseline Characteristics

The baseline characteristics of 13,255 participants are summarized in Table 1 according to whether they have NAFLD. Compared with non-NAFLD participants, the NAFLD group members were older, more likely to be men, and tended to have a higher BMI, total energy intake and ALT, AST, TC, and TG levels, and lower HDL-C levels. There was no difference in LDL-C levels between the two groups.

### 3.3. Dietary Niacin Intake and NAFLD

**Figure 2:**
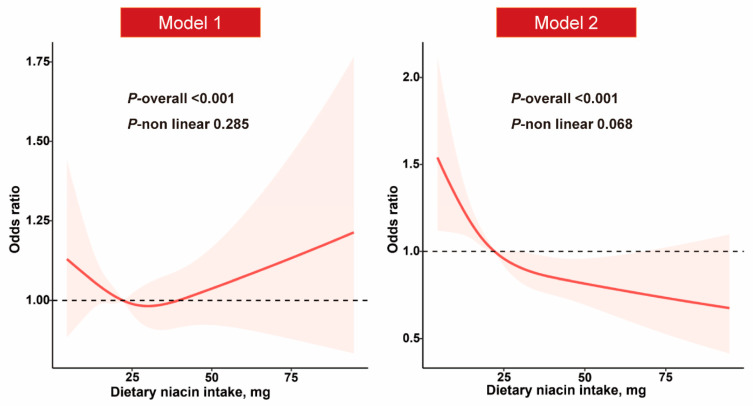
Relationship between dietary niacin intake and NAFLD.

Following multivariable adjustment in Model 2, there is no evident nonlinear relationship between dietary niacin intake and the presence of NAFLD in the restricted cubic spline regression (*p*_overall_ < 0.001; *p*_nonlinear_ = 0.068) (Figure 2). In Model 1, the ORs and 95% CIs from the lowest to highest categories of dietary niacin intake were 1.00 (reference), 0.88 (0.75–1.04), 0.92 (0.79–1.07), and 0.93 (0.79–1.09), respectively, for NAFLD (*p*_trend_ = 0.469). The multivariable-adjusted ORs and 95% CIs for NAFLD in Model 2 were 0.84 (0.68–1.03), 0.80 (0.65–0.97), and 0.69 (0.55–0.85), respectively, when comparing the second, third, and fourth quartiles of niacin intake levels to the lowest quartile (*p*_trend_ = 0.001) (Table 2).

Model 1: adjusted for age and gender. Model 2: further adjusted for race/ethnicity, education levels, family income–poverty ratio, smoking status, physical activity, body mass index, total energy intake, hypertension, high cholesterol, and diabetes.

**Table 2:** Association between daily niacin intake and NAFLD.

	Dietary Niacin Intake, mg				
	Quartile 1 (≤16.3)	Quartile 2 (16.4–22.1)	Quartile 3 (22.2–29.1)	Quartile 4 (≥29.2)	*p*_trend_
Model 1	1.00 (Ref)	0.88 (0.75, 1.04)	0.92 (0.79, 1.07)	0.93 (0.79, 1.09)	0.597
Model 2	1.00 (Ref)	0.84 (0.68, 1.03)	0.80 (0.65, 0.97)	0.69 (0.55, 0.85)	0.001

Model 1: adjusted for age and gender; Model 2: further adjusted for race/ethnicity, education levels, family income–poverty ratio, smoking status, physical activity, body mass index, total energy intake, hypertension, high cholesterol, and diabetes.

Moreover, the associations between daily niacin intake according to the Recommended Dietary Allowance (RDA) and Tolerable Upper Intake Level (UL) standards, and NAFLD in male and female populations were also examined (Supplementary Table S1). The RDA of niacin is 16 mg/day for men and 14 mg/day for women [22], and the UL of niacin is 35 mg/day for both men and women [23]. We found that compared with the reference group (16–35 mg/day), the multivariate-adjusted ORs and 95% CIs for NAFLD were 1.40 (0.76–2.55) and 0.87 (0.58–1.32) in the lowest niacin intake group (<16 mg/day) and the highest niacin intake group (>35 mg/day), respectively. In women, compared with the reference group (14–35 mg/day), the multivariate-adjusted ORs and 95% CIs for NAFLD were 1.92 (1.18–3.15) and 0.87 (0.45–1.66) in the lowest niacin intake group (<14 mg/day) and the highest niacin intake group (>35 mg/day).

### 3.4. Stratified Analyses

We found a significant interaction between dietary niacin intake and hypertension with the presence of NAFLD (*p*_interaction_ = 0.033) (Table 3). In the subgroup with hypertension compared with the reference group, the OR and 95% CI for NAFLD was 0.96 (0.64–1.44) in the fourth quartile, and in the subgroup without hypertension compared with the reference group, the OR and 95% CI for NAFLD was 0.54 (0.40–0.74) in the fourth quartile. However, no significant interactions were found between dietary niacin intake and any of the other strata variables with NAFLD.

In addition to the above results, consistent results were also demonstrated when dietary niacin intake levels were categorized into quintiles (Supplementary Table S2).

## 4. Discussion

In this nationally representative US population-based study, our study is the first to identify an inverse association between daily niacin consumption and NAFLD. Participants with a niacin intake greater than 22.2 mg/day (quartiles 3 and 4) had a significantly lower risk of NAFLD compared to those with niacin intake of less than 16.4 mg/day (quartile 1). A significant interaction between dietary niacin intake and hypertension with the presence of NAFLD was also found.

According to an animal study in rats, the inclusion of niacin at doses of 0.5% and 1.0% (approximately 0.1 g and 0.2 g daily) in a high-fat diet can effectively decrease liver fat content, liver weight, and liver oxidation products, and prevent hepatic steatosis [11]. Ye et al. also demonstrated that the hepatic lipogenesis inhibition in C57BL/6 mice can be achieved by niacin (50 mM, dissolved in water), and the finding was in line with mechanistic investigations conducted on HepG2 cells [24]. The above results indicate that niacin exhibits significant efficacy in preventing the occurrence and regression of experimental hepatic steatosis. However, Fang et al. found that niacin administration at a daily dose of approximately 360 mg/kg for a duration of 20 weeks promotes the development of diet-induced hepatic steatosis in B6129 mice [25]. In addition, there are also some studies showing the potential benefits of niacin in humans. A 23-week intervention trial involving 39 participants with hypertriglyceridemia revealed that niacin effectively reduced liver fat content by 47.2% [26]. Similarly, Pirinen et al. found that niacin is an effective NAD^+^ booster in humans, resulting in enhanced muscle strength and reduced fatty liver in individuals with mitochondrial myopathy [12]. Apart from the evidence from animal and human studies, the preclinical research also discovered that niacin can inhibit and reverse hepatic steatosis and inflammation, while also acting as a preventive measure against fibrosis. These effects are accomplished by means of diminishing oxidative stress, impeding the activity of diacylglycerol acyltransferase 2 (DGAT2) and NADPH oxidase, as well as through various other potential mechanisms [27,28]. The results of our study were comparable to these previous studies. We observed a positive effect of increased dietary niacin intake on NAFLD after controlling for multiple confounders. Specifically, a higher intake of dietary niacin was associated with a 20% (22.2–29.1 mg/day vs. ≤16.3 mg/day) and 31% (≥29.2 mg/day vs. ≤16.3 mg/day) reduction in the risk of NAFLD among the studied population. Although no significant inverse association between dietary niacin and NAFLD was found in Model 1 (adjusted for age and gender only), this may be due to insufficient adjustment for potential confounders.

In addition, the associations between daily niacin intake according to the RDA and UL standards and NAFLD in male and female populations indicated that in women, consuming less than the RDA of niacin was associated with a 92% increased risk of NAFLD compared to the levels between the RDA and UL, while this was not observed in men. For both men and women, dietary niacin intakes above the UL of niacin were not found to increase or decrease the risk of NAFLD compared to those within the RDA-UL range. The dose–response relationship between dietary niacin levels and the risk of NAFLD needs to be further explored in different cohort populations.

In our analysis, we also identified a noteworthy interaction between dietary niacin intake and hypertension. This finding suggests that the impact of dietary niacin intake on NAFLD could vary based on the existence of hypertension. It seems that in individuals without hypertension, higher niacin intake levels are more strongly associated with a lower risk of NAFLD. Due to the bidirectional relationship between hypertension and NAFLD indicated in previous studies [29,30,31], and the cross-sectional nature of our research, the precise mechanism by which hypertension impacts the association remains unclear. Prospective studies are needed to further determine whether the protective effect of higher niacin intake on the risk of NAFLD is different in individuals with and without hypertension.

Several studies reported that a variety of pathological conditions, such as cardiovascular disease, obesity, and neurodegenerative diseases, are related to dysregulation of cellular NAD^+^ levels [32,33,34]. In the development of NAFLD, there is an accumulation of hepatic steatosis and a decrease in liver NAD^+^ levels [35]. Moreover, Zhou et al. demonstrated that a deficiency in NAD^+^ associated with aging is a significant risk factor for NAFLD [10]. Niacin is one of the precursors of NAD^+^, and the basic requirement for NAD^+^ synthesis can be fulfilled either through dietary tryptophan or by consuming less than 20 mg of daily niacin [36]. While the exact molecular mechanism of the association between dietary niacin intake and the risk of NAFLD requires further investigation, our findings align with existing evidence and are biologically plausible.

One of the strengths of this study is that we ensured the inclusion of a nationally representative sample of study population in the United States by strictly adhering to inclusion and exclusion criteria, which enhanced the generalizability of our results. Additionally, our study spanned a remarkable duration of 16 years, covering 8 cycles, making it the largest-scale study to date on the association between dietary niacin intake and NAFLD, which filled the gap regarding the relationship between dietary niacin and NAFLD. However, our study has several limitations that should be considered. First, our research design is of an observational nature. This type of design solely permits the establishment of epidemiological associations rather than conclusive causal inferences. Nonetheless, it possesses the capability to furnish valuable insights for subsequent mechanistic investigations. Second, we may not have been able to adjust for all potential confounders, which leaves open the possibility of residuals and unknown confounders that cannot be entirely ruled out. In addition, although dietary data were obtained through two 24 h dietary recalls, the existence of recall bias could not be completely ruled out.

## 5. Conclusions

In conclusion, our study indicates that higher dietary niacin intake is associated with a lower chance of developing NAFLD. The dose–response relationship of dietary niacin intake in reducing the presence of NAFLD needs to be further investigated in prospective studies to determine optimal intake levels.

## Figures and Tables

**Table 1:** Characteristics of participants with or without NAFLD in NHANES from 2003 to 2018.

Characteristic	Overall N = 13,255	Non-NAFLD N = 8877	NAFLD N = 4378	*p* Value
Age, years	48.0 (34.0, 61.0)	45.0 (31.0, 59.0)	54.0 (39.0, 65.0)	<0.001
Age (years), n (%)				<0.001
≤39	4145 (35.6)	3212 (40.5)	933 (25.1)	
40–59	4357 (36.7)	2833 (35.4)	1524 (39.4)	
≥60	4753 (27.7)	2832 (24.1)	1921 (35.5)	
Gender, n (%)				<0.001
Male	6238 (47.0)	3912 (43.8)	2326 (53.8)	
Female	7017 (53.0)	4965 (56.2)	2052 (46.2)	
Race/ethnicity, n (%)				<0.001
Mexican American	2170 (8.5)	1099 (6.3)	1071 (13.1)	
Non-Hispanic white	5992 (68.1)	3948 (67.4)	2044 (69.4)	
Non-Hispanic black	2557 (10.3)	2061 (12.3)	496 (6.1)	
Other	2536 (13.2)	1769 (13.9)	767 (11.5)	
Education levels, n (%)				<0.001
<High school	3167 (15.8)	1836 (13.6)	1331 (20.5)	
High school	3011 (23.3)	2009 (22.9)	1002 (24.3)	
Some college or above	7068 (60.8)	5026 (63.4)	2042 (55.1)	
Family income–poverty ratio, n (%)				0.002
<1.0	2249 (13.0)	1448 (12.4)	801 (14.3)	
1.0–3.0	5280 (37.5)	3441 (36.4)	1839 (39.8)	
>3.0	4691 (49.6)	3331 (51.2)	1360 (46.0)	
Smoking status, n (%)				<0.001
Never smoker	7586 (56.9)	5289 (59.4)	2297 (51.5)	
Former smoker	3370 (25.3)	1989 (22.4)	1381 (31.6)	
Current smoker	2295 (17.8)	1597 (18.2)	698 (16.9)	
Physical activity, n (%)				<0.001
Active	4100 (62.1)	3102 (65.1)	998 (53.4)	
Inactive	2695 (37.9)	1874 (34.9)	821 (46.6)	
BMI, kg/m^2^	27.9 (24.1, 32.6)	25.9 (23.0, 29.2)	33.6 (29.9, 38.5)	<0.001
BMI (kg/m^2^), n (%)				<0.001
<30.0	8097 (62.5)	6849 (79.5)	1248 (25.3)	
≥30.0	5130 (37.5)	2015 (20.5)	3115 (74.7)	
Total energy, kcal	1963.4 (1533.2, 2496.9)	1939.5 (1524.0, 2464.5)	2018.4 (1550.5, 2569.2)	0.004
Hypertension, n (%)				<0.001
Yes	4893 (32.9)	2640 (25.0)	2253 (50.2)	
No	8337 (67.1)	6218 (75.0)	2119 (49.8)	
High cholesterol, n (%)				<0.001
Yes	4666 (39.3)	2702 (34.4)	1964 (49.5)	
No	6756 (60.7)	4820 (65.6)	1936 (50.5)	
Diabetes, n (%)				<0.001
Yes	1761 (9.9)	678 (4.9)	1083 (20.7)	
No	11,486 (90.1)	8194 (95.1)	3292 (79.3)	
ALT, U/L	21.0 (16.0, 28.0)	19.0 (15.9, 25.0)	26.0 (20.0, 35.1)	<0.001
AST, U/L	23.0 (19.0, 27.0)	22.0 (19.0, 26.0)	24.1 (21.0, 29.0)	<0.001
TC, mg/dL	190.0 (164.0, 218.0)	189.0 (164.0, 216.0)	192.0 (165.0, 221.0)	0.033
TG, mg/dL	105.0 (72.0, 155.0)	90.0 (64.0, 127.0)	148.0 (105.0, 209.0)	<0.001
LDL-C, mg/dL	111.0 (90.0, 136.0)	111.0 (90.0, 135.0)	113.0 (90.0, 138.0)	0.157
HDL-C, mg/dL	51.0 (42.0, 62.0)	55.0 (46.0, 66.0)	44.0 (38.0, 52.0)	<0.001

Abbreviations: NAFLD, nonalcoholic fatty liver disease; NHANES, National Health and Nutrition Examination Survey; BMI, body mass index; ALT, alanine aminotransferase; AST, asparate aminotransferase; TC, total cholesterol; TG, triglyceride; LDL-C, low-density lipoprotein cholesterol; HDL-C, high-density lipoprotein cholesterol.

**Table 3:** Associations of dietary niacin intake with NAFLD in various subgroups in NHANES 2003–2018.

	Dietary Niacin Intake, mg				
Characteristic	Quartile 1 (≤16.3)	Quartile 2 (16.4–22.1)	Quartile 3 (22.2–29.1)	Quartile 4 (≥29.2)	*p*_interaction_
Age, years					
<60	1.00 (Ref)	0.81 (0.62, 1.05)	0.86 (0.66, 1.12)	0.71 (0.54, 0.94)	0.654
≥60	1.00 (Ref)	0.88 (0.66, 1.19)	0.72 (0.54, 0.96)	0.62 (0.42, 0.93)	
Gender					
Male	1.00 (Ref)	0.73 (0.50, 1.08)	0.69 (0.49, 0.99)	0.61 (0.43, 0.88)	0.615
Female	1.00 (Ref)	0.83 (0.65, 1.06)	0.80 (0.62, 1.05)	0.70 (0.50, 0.98)	
Race/ethnicity					
Non-Hispanic white	1.00 (Ref)	0.70 (0.53, 0.94)	0.71 (0.54, 0.94)	0.63 (0.47, 0.83)	0.294
Other	1.00 (Ref)	1.05 (0.80, 1.38)	0.99 (0.75, 1.30)	0.82 (0.60, 1.13)	
Education levels					
≤High school	1.00 (Ref)	0.89 (0.67, 1.20)	0.91 (0.67, 1.23)	0.78 (0.56, 1.07)	0.961
Some college or above	1.00 (Ref)	0.78 (0.59, 1.05)	0.73 (0.54, 0.98)	0.63 (0.45, 0.89)	
Family income–poverty ratio					
≤3.0	1.00 (Ref)	0.98 (0.78, 1.22)	0.97 (0.76, 1.24)	0.86 (0.65, 1.13)	0.554
>3.0	1.00 (Ref)	0.64 (0.45, 0.93)	0.61 (0.42, 0.89)	0.51 (0.35, 0.72)	
Smoking status					
Never	1.00 (Ref)	0.75 (0.56, 1.00)	0.82 (0.59, 1.14)	0.57 (0.41, 0.80)	0.227
Former or current	1.00 (Ref)	0.95 (0.71, 1.29)	0.77 (0.55, 1.06)	0.83 (0.60, 1.15)	
Physical activity					
Active	1.00 (Ref)	0.85 (0.63, 1.15)	0.80 (0.58, 1.09)	0.61 (0.43, 0.85)	0.315
Inactive	1.00 (Ref)	0.82 (0.60, 1.11)	0.82 (0.59, 1.14)	0.82 (0.58, 1.16)	
BMI					
<30.0	1.00 (Ref)	0.95 (0.70, 1.29)	0.80 (0.58, 1.10)	0.77 (0.56, 1.08)	0.413
≥30.0	1.00 (Ref)	0.76 (0.58, 0.99)	0.81 (0.62, 1.07)	0.65 (0.48, 0.88)	
Hypertension					
Yes	1.00 (Ref)	0.99 (0.72, 1.37)	1.02 (0.74, 1.41)	0.96 (0.64, 1.44)	0.033
No	1.00 (Ref)	0.70 (0.52, 0.94)	0.66 (0.50, 0.88)	0.54 (0.40, 0.74)	
High cholesterol					
Yes	1.00 (Ref)	0.84 (0.63, 1.13)	0.76 (0.56, 1.02)	0.58 (0.40, 0.85)	0.876
No	1.00 (Ref)	0.86 (0.65, 1.13)	0.87 (0.66, 1.14)	0.82 (0.60, 1.11)	
Diabetes					
Yes	1.00 (Ref)	0.61 (0.38, 1.00)	0.76 (0.49, 1.19)	0.88 (0.48, 1.62)	0.552
No	1.00 (Ref)	0.87 (0.69, 1.10)	0.81 (0.65, 1.01)	0.67 (0.53, 0.85)	

Abbreviations: NAFLD, nonalcoholic fatty liver disease; BMI, body mass index; NHANES, National Health and Nutrition Examination Survey. Data are presented as OR (95% CI). Adjusted for age, gender, race/ethnicity, education levels, family income–poverty ratio, smoking status, physical activity, body mass index, total energy intake, hypertension, high cholesterol, and diabetes. The strata variable was not included when stratifying by itself.

## Data Availability

The data described in the manuscript, codebook, and analytic code will not be made available as they was obtained from the NHANES database, which is freely accessible to all researchers worldwide.

## Supplementary Materials

The following supporting information can be downloaded at: https://www.mdpi.com/article/10.3390/nu15194128/s1, Table S1: Association between daily niacin intake according to RDA and UL standards and NAFLD. Table S2: Association between daily niacin intake and NAFLD.
