# Supplementary material for: Association between Dietary Niacin Intake and Nonalcoholic Fatty Liver Disease: NHANES 2003–2018
Source: Nutrients. 2023 Sep 25;15(19):4128. doi: 10.3390/nu15194128 (PMC10574350; doi:10.3390/nu15194128)
Supplement: Supplementary file 1 [file nutrients-15-04128-s001.zip › Supplementary Table S1.pdf]

Supplementary Table S1. Association between daily niacin intake according to RDA and UL standards and NAFLD

	Dietary niacin intake, mg [Men]			Dietary niacin intake, mg [Women]		
	< 16	16-35	> 35	< 14	14-35	> 35
Model 1	1.28 (1.00, 1.64)	1.00 (Ref)	0.93 (0.76, 1.14)	1.15 (0.95, 1.40)	1.00 (Ref)	0.99 (0.71, 1.40)
Model 2	1.40 (0.76, 2.55)	1.00 (Ref)	0.87 (0.58, 1.32)	1.92 (1.18, 3.15)	1.00 (Ref)	0.87 (0.45, 1.66)

Abbreviations: NAFLD, nonalcoholic fatty liver disease; RDA, recommended dietary allowance; UL, Tolerable Upper Intake Level

Model 1: crude model

Model 2: further adjusted for race / ethnicity, education, family income–poverty ratio, smoking status, physical activity, body mass index, total energy intake, hypertension, high cholesterol, and diabetes.
