# Supplementary material for: Association between Dietary Niacin Intake and Nonalcoholic Fatty Liver Disease: NHANES 2003–2018
Source: Nutrients. 2023 Sep 25;15(19):4128. doi: 10.3390/nu15194128 (PMC10574350; doi:10.3390/nu15194128)
Supplement: Supplementary file 1 [file nutrients-15-04128-s001.zip › Supplementary Table S2.pdf]

Supplementary Table S2. Association between daily niacin intake and NAFLD

	Dietary niacin intake, mg					
	Quintile 1 (≤ 15.2)	Quintile 2 (15.3-19.7)	Quintile 3 (19.8-24.5)	Quintile 4 (24.6-31.2)	Quintile 5 (≥ 31.3)	P_{trend}
Model 1	1.00 (Ref)	1.00 (0.82, 1.22)	1.06 (0.86, 1.31)	0.92 (0.78, 1.09)	1.12 (0.92, 1.37)	0.271
Model 2	1.00 (Ref)	0.97 (0.80, 1.18)	1.00 (0.81, 1.23)	0.84 (0.71, 0.99)	1.01 (0.84, 1.23)	0.948
Model 3	1.00 (Ref)	0.87 (0.68, 1.13)	0.89 (0.70, 1.12)	0.73 (0.57, 0.94)	0.76 (0.59, 0.98)	0.027

Model 1: crude model

Model 2: adjusted for age and gender

Model 3: further adjusted for race / ethnicity, education levels, family income–poverty ratio, smoking status, physical activity, body mass index, total energy intake, hypertension, high cholesterol, and diabetes.
